# Prevalence of mental disorders in prisons in the UK: a systematic review and meta-analysis

**DOI:** 10.1192/bjo.2021.755

**Published:** 2021-06-18

**Authors:** Nivedita Rebbapragada, Vivek Furtado, George William Hawker-Bond

**Affiliations:** Warwick Medical School

## Abstract

**Aims:**

To report pooled prevalence of all mental disorders among the general prison population in the United Kingdom (UK). This includes individuals in Young Offender Institutions (YOI), youth custody and adult prisons across all categories. A secondary aim explores possible sources of heterogeneity by performing subgroup and meta-regression analysis across certain covariates (e.g. sex of prisoner). We hypothesise that contemporary estimates of mental disorders are higher than the general population.

**Background:**

Prevalence of mental health problems among prisoners are considerably higher than the general population; this poses an important public health concern. Individuals who require diversion to appropriate psychiatric services are becoming embroiled in the revolving door of the criminal justice system. However, there are no up-to-date reviews assessing prevalence of mental disorders across the general prison population in the UK. This study aims to address this gap.

**Method:**

We conducted a systematic search of PsycINFO (1923 – October 2019), MEDLINE (1946 – October 2019), EMBASE (1947 – October 2019) and Web of Science (all years) of articles reporting prevalence of mental disorders in UK prison populations (PROSPERO registration number: CRD42019132685). The Joanna Briggs Institute (JBI) Appraisal Checklist for Studies Reporting Prevalence Data assessed study quality and bias. Pooled prevalence of each mental disorder was calculated using Stata statistical software 16.0 via the metaprop command. Forest plots present prevalence estimates with study weights and associated 95% confidence intervals (CI). Overall, 20 studies satisfied inclusion criteria, comprising of 12,335 prisoners across England, Wales and Scotland.

**Result:**

We identified higher rates of neurotic disorders (28.9%, 95% CI 0.71–74.7%), personality disorders (23.5%, 95% CI 13.6–35.2%), alcohol (22.7%, 95% CI 12.2–35.1%) and drug dependence (26.7%, 95% CI 15.0–40.4%). The lowest prevalence rates included schizophrenia (2.42%, 95% CI 0.78–4.84%), panic disorders (3.88%, 95% CI 3.17% – 4.64%), adjustment disorders (3.83%, 95% CI 1.19–7.84%) and intellectual disability (2.90%, 95% CI 0.90–5.80%). Meta-regressions for psychotic disorder and personality disorder revealed no significant differences across study year, sample size and gender.

**Conclusion:**

Our prevalence estimates of mental disorders in prisons are higher than the general English population. However, we should acknowledge the influence of considerable heterogeneity. These findings demonstrate the need to quantify current prevalence of mental disorders amongst prisoners in the UK. We recommend for the government to consider performing an up-to-date census of psychiatric morbidity to facilitate service provision.

